# Evaluating the Surgeon’s Experience as a Risk Factor for Post-Esophagectomy Chylothorax on a Four-Year Cohort

**DOI:** 10.7759/cureus.8696

**Published:** 2020-06-19

**Authors:** Nadim Malibary, Simone Manfredelli, Abdullah Almuttawa, John-Baptiste Delhorme, Benoit Romain, Cecile Brigand, Serge Rohr

**Affiliations:** 1 Surgery, King Abdulaziz University, Jeddah, SAU; 2 Visceral and General Surgery, Hautepierre Hospital, Strasbourg, FRA; 3 Visceral and Digestive Surgery, Hautepierre Hospital, Strasbourg, FRA; 4 Surgery, University of Jeddah, Jeddah, SAU; 5 Visceral and Digestive Surgery, Hautepierre University Hospital, Strasbourg, FRA

**Keywords:** chylothorax, esophagectomy, surgical training, complications, esophageal cancer

## Abstract

Background: Chylothorax (CHT) is a known post-operative complication after esophageal surgery with vaguely defined risk factors.

Methods: This is a retrospective chart review of 70 consecutive patients with operable cancer over a period of four years (January 2013 to December 2016). Ivor Lewis and McKeown interventions were performed. Thoracic duct is identified and ligated routinely. Factors related to the patient, the tumor, and the operating surgeon were analyzed.

Results: Incidence of CHT was 10%. Surgeons with less than five years of esophageal surgery experience had the most CHT, 71% (p=0.001). No association was found between tumor location, type, body mass index (BMI), neoadjuvant therapy, response to neoadjuvant therapy or male sex, and CHT. The odds of developing CHT were 17 times higher in patients operated by a junior surgeon (odds ratio, OR=17.67, confidence interval, CI 2.68-116.34, p=0.003). Four patients (5.7%) had anastomotic leaks, none of them had CHT. Senior surgeons had less operative time and harvested more lymph nodes (p=0.0002 and p=0.1086 respectively).

Conclusion: Surgeon’s experience might be considered a major risk factor to develop CHT. This finding needs to be confirmed by a larger multicentric series taking into consideration the human factor.

## Introduction

Chylothorax (CHT) is the accumulation of lymphatic fluid in the pleural cavity. Its incidence after esophageal operations varies between 0.9% and 11.6% in the literature [1-3]. Different studies have discussed and researched the risk factors for developing CHT after esophageal and cardiopulmonary interventions including factors related to the patient, their treatment regimen, and the tumor [4-5]. Esophageal surgery remains technically challenging and requires a certain set of skills and experience. For that, the centralization of esophagectomies has helped to improve outcomes [6]. Between post-operative complications, CHT is an important entity, although it has relatively low incidence rates. Its treatment is usually lengthy and exhaustive for the patient and might lead to a second or third intervention. Methods of treatment start with fasting in addition to the modification of enteral or parenteral feeding using medium-chain triglyceride products. If this is not sufficient, various other solutions exist, ranging from octeroid agonists, radiological embolization of the thoracic duct, and as a last solution, a reoperation to religate the thoracic duct [1, 4].

Our work was concentrated on post-esophagectomy CHT in adults and its associated risk factors, both preoperatively and intraoperatively. Factors related to the patient and the tumor itself were considered.

## Materials and methods

Patient selection

We retrospectively analyzed 70 consecutive patients over a period of four years (January 2013 to December 2016) at a university general surgery department in a regional referral center with more than 40 esophageal interventions per year (Strasbourg University Hospital - Hospital of Hautepierre). All patients had resectable cancers. Two types of operation were performed: Ivor Lewis and McKeown (see Table 1). Among the different factors analyzed in Table 1, we have focused our analysis on the surgeon‘s experience. Surgeons with fewer than five years of experience of esophageal surgery were categorized as “junior surgeons” and those with more than five years of experience were categorized as “senior surgeons.” We considered the response to neoadjuvant therapy as the regression of the tumor on gastroscopy or CT scan, or improvement of dysphagia. Thirty-day postoperative complications were evaluated according to the Dindo-Clavien classification [7].

**Table 1:** Baseline demographics and clinical characteristics. IQR: Interquartile range; BMI: Body Mass Index

	Total populations
Age (median, IQR)	61.5 (54-67)
Male sex (n, %)	56 (80%)
BMI >25 (n, %)	42 (60%)
Ivor Lewis	63 (90%)
Tumor location in the lower third	64 (91.42%)
Cancer type	
Squamous cell	7 (10%)
Adenocarcinoma	63 (90%)
Operations by a junior surgeon	12 (17.14%)
Neoadjuvant therapy	
No therapy	9 (12.86%)
Chemotherapy	43 (61.43%)
Radio-chemotherapy	18 (25.71%)
Response to neoadjuvant therapy	49 (70%)
Preoperative weight loss (>10% of total body weight)	23 (32.86%)
Median length of stay	16.5 (13-23)
Number of harvested lymph nodes per intervention	25.7 (11.18)
Operative time in minutes	356 (80.09)

Surgical technique

The operative technique is standardized and performed in the same manner by all operators. Selective intubation is performed in all patients as well as a thoracic epidural for post-operative analgesia. We briefly describe the two procedures (Ivor Lewis and McKeown) knowing that they share a similar thoracic and abdominal approach [1, 8-9].

Ivor Lewis: a two-stage operation with a two-field lymphadenectomy. Gastric mobilization and Kocher manoeuver is the first step and could be performed by laparotomy or laparoscopy. Then, a right-sided posterolateral thoracotomy is performed in the fifth intercostal space. The azygos vein is resected, followed by a monobloc resection of the esophagus and the thoracic duct; the latter is ligated at the level of the thoracic outlet using a multifilament thread. We pull the stomach to the thoracic cavity, complete the resection, and perform a mechanic terminolateral esogastric anastomosis using a circular stapler. Two thoracic drains are left in the pleural cavity.

McKeown intervention: a three-stage operation. Abdominal and thoracic steps are generally similar to the previous intervention but in addition there is a left cervical incision where we extract the esophagus and perform a manual terminolateral esogastric anastomosis.

An anastomotic solidarity test by Blue of Methylene is performed at the end by means of a nasogastric tube.

Diagnosis of chylothorax

Once the thoracic drainage became milky or more than 500 cc was produced per day, fluid analysis was performed searching for chylomicrons. We considered the presence of chylomicrons (Hydragel lipo test, SEBIA) as diagnostic [10]. No provocation tests were performed to confirm the diagnosis.

Statistical analysis

Microsoft Excel was used to collect and code the data. Statistical analysis was undertaken using StataCorp. 2013 (Stata Statistical Software, release 13. StataCorp LP, College Station, TX).

Baseline demographics, clinical, and laboratory data were summarized in the form of averages [median, IQR (inter-quartile range)], ± standard deviation for continuous variables, and percentages for categorical variables. This summary was presented for the total study population and then by whether or not the patient was diagnosed with CHT. Comparison between patient groups was performed using the student’s t-test for the continuous variables and the Fisher’s exact test for the categorical variables. To identify the predictors of CHT, we used that variable as an outcome against various predictor variables. Logistic regression was used to examine this relationship. Variables were included in the multivariate model if their p-value was <= 0.10. The odds ratio (OR), p-values, and 95% confidence intervals (CIs) are presented.

For all of the regression models and statistical tests, a p-value of less than 0.05 was considered statistically significant.

## Results

Evaluation of risk factors of chylothorax

The incidence of CHT in our series was 10%. We divided the cohort into two groups: Group A comprised patients without CHT and Group B included patients with CHT. Univariate analysis (Table 2) showed that the tumor location at the lower third of the esophagus and adenocarcinoma was associated with 71% of Group B (p=0.107 and p=0.14 respectively). Junior surgeons were a significant risk factor for CHT (p=0.001). The other factors did not differ between the two groups.

**Table 2:** Comparison between chylothorax and nonchylothorax groups. IQR: Interquartile range, BMI: Body Mass Index

	No chylothorax Group A n= 63 (90%)	Chylothorax Group B n=7 (10%)	p-value
Age (median, IQR)	61 (54-67)	64 (51-72)	0.84
Male sex (n, %)	52 (82.54%)	4 (57.14%)	0.14
BMI >25 (n, %)	37 (58.73%)	5 (71.43%)	0.69
Ivor Lewis intervention	57 (90.48%)	6 (85.71%)	0.54
Tumor in the lower third	59 (93.65%)	5 (71.43%)	0.107
Type of cancer			
Squamous cell	5 (7.94%)	2 (28.57%)	0.14
Adenocarcinoma	58 (92.06%)	5 (71.43%)	
Interventions by a junior surgeon	7 (11.11%)	5 (71.43%)	0.001
Preoperative therapy			
No therapy	7 (11.11%)	2 (28.57%)	0.29
Chemotherapy	40 (63.49%)	3 (46.86%)	
Radio-chemotherapy	16 (25.40%)	2 (28.57%)	
Response to neoadjuvant therapy	45 (71.43%)	4 (57.14%)	0.42
Preoperative weight loss (>10% of total body weight)	21 (33.33%)	2 (28.57%)	1.00
Median length of stay	16 (13-22)	26 (15-96)	0.0002
Harvested lymph nodes	26.05 (11.34)	22.57 (9.74)	0.47
Operative time	346 (71.05)	446 (104.64)	0.0012

The median length of stay was 16 days for Group A and 26 days for Group B (p=0.0002).

There were four anastomotic leaks (5.7%) but none had a concomitant CHT. Thirty-one patients had a postoperative complication (44.2%). There were 20 complications of grades I-II (15 pneumonia, three cardiac arrhythmias, one post-operative ileus, one left vocal cord paralysis), and 16 complications of grades III-IV (four pyloric spasms, one pulmonary embolism, one splenic infarction, one adult respiratory distress syndrome, one hemorrhagic cerebral metastasis, four pneumonia, one anastomotic stenosis, two pancreatic fistulas, one diaphragmatic hernia). There was no mortality during the first 30 post-operative days. There was no significant difference in the complications of the two groups. Two patients in Group B were treated surgically for highly productive chest drains >2 L per day associated with respiratory distress, three were treated successfully by conservative measures, while two had additional radiological embolization of the thoracic duct.

Is the surgeon’s experience an independent risk factor of chylothorax?

Twelve interventions were performed by junior surgeons (17%), of which five (42%) developed CHT (p=0.001). Senior surgeons had two CHT (3%).

Adjusting for sex, type of cancer and tumour location, the odds of developing CHT were 17 times higher in patients operated on by a junior surgeon compared to those operated on by a senior surgeon (OR=17.67, CI 2.68-116.34, p=0.003) (Table 3).

**Table 3:** Multivariate analysis. OR: Odds ratio; CI: Confidence interval

	OR (95%CI)	p-value
Male sex	0.42 (0.06-2.81)	0.370
Adenocarcinoma	0.21 (0.01-3.12)	0.254
Tumors in the lower third	0.40 (0.05-3.49)	0.408
Junior surgeon	17.67 (2.68-116.34)	0.003

When comparing operative time (OT) between the senior and junior surgeons, senior surgeons had an average OT of 338.8 minutes, whereas for junior surgeons it was 440 minutes (p=0.0002). Senior surgeons harvested more lymph nodes (22.67 vs. 21, p=0.1086) but no difference was observed in terms of hospital stay duration (22.9 vs. 22.8 days, p=0.9842) (see Table 4).

**Table 4:** Comparison between interventions done by a senior and junior surgeon.

	Senior surgeon (N=58)	Junior surgeon (N=12)	p-value
Operative time	338.81 (71.81)	440.08 (65.17)	0.0002
Number of harvested lymph nodes	26.67 (11.17)	21 (10.41)	0.1086
Hospital stay	22.86 (23.41)	22.75 (23.41)	0.9842
Chylothorax	2 (3.45%)	5 (41.67%)	0.001

## Discussion

Chylothorax, according to Schild [4], is ‘a collection of chyle in the pleural cavity resulting from leakage from the lymphatic vessels,’ most probably the thoracic duct. Many situations can precipitate it such as cardio-thoracic surgery, trauma, or interventional radiology. The thoracic duct drains the lymph below the diaphragm. Its close proximity to the esophagus and aorta is the main risk of intraoperative injury because that area is dissected during esophageal interventions. Its incidence after esophageal surgery varies between 0.9% and 11.6% [1-3].

Although there are no clear guidelines for treating CHT [11], in our practice, once the thoracic drainage becomes highly productive (>0.5-1 L per day) or becomes creamy regardless of the volume, pleural fluid analysis is performed looking for chylomicrons. We stop prophylactically the oral intake, switch both enteral and parenteral feeding to low chain lipids, and start patients simultaneously on octeroid injections. We do not perform any provocative tests to confirm the diagnosis. Reduction of drain volume or loss of the creamy aspect were indirect diagnostic signs.

Preserving the thoracic duct is technically difficult and no general consensus has been established either for resecting or preserving it [1]. Variations exist between studies regarding the benefit of ligation for the prevention of CHT [12-13] but there is no direct oncological benefit from thoracic duct resection aside from significantly more lymph nodes harvested, as with azygos vein resection. In two studies, there was no difference in the incidence of CHT between the resected and preserved group [1, 14]. Some studies showed that when ligating the thoracic duct, we might get less pleural effusion and CHT, which leads to less thoracic complications, notably pneumonia and sepsis [1, 4, 15-16].

Some articles compared different methods of ligation, either by threads, clips, or sealing devices [15, 17], with one study preferring the clip applier [15] with no significant difference between any of the methods. In our department, we perform routine thoracic duct ligation by means of multifilament threads, sometimes reinforced with metallic clips.

Various papers have studied the risk factors (RFs) associated with CHT. Shah et al. [18] found that it was associated with squamous cell carcinoma. Other risk factors include tumors located in the middle third of the esophagus [5, 19] and positive lymph nodes [20]. Cervical anastomosis and neoadjuvant treatments were also considered as RFs [3]. In another study, there was no influence of age, gender, number of lymph nodes harvested, N status in the TNM classification, R status, or pathological grade [3].

Miao et al. [21] suggested a body mass index (BMI) of <25 as an independent RF in one study, while in another he found that a BMI >25 was protective [22], probably because this made it easier to visualize the duct, although this was contradicted by another author [11]. In our cohort, a BMI of more than 25 had no effect on CHT (p=0.69). No statistically significant difference was found between the minimally invasive approach and thoracotomy [23-24], or between the transhiatal and transthoracic approaches [3]. In another contradictory paper, they stated that ligation was preventive, while minimally invasive surgery led to more CHT [9].

Gupta et al. [5] found that patients with complete response to neoadjuvant treatment had reduced CHT, while middle third tumors had less. In our patients, the response to preoperative therapy was not statistically significant (p=0.42), while lower esophageal tumors had a better correlation (p=0.107).

Furthermore, in our research we found one study mentioning the human factor as being a predisposal to CHT but all patients were operated by two surgeons with similar techniques with no difference in post-operative morbidity. The statistical analysis was controlled for this confounding factor [11]. Another paper stated that all surgeries were performed by senior consultants [5].

In our series, inexperienced surgeons were mentored by senior consultants and the critical part of the intervention, namely the anastomosis, is always performed with their help. Over four years, we had just four anastomotic leaks (5.7%) which affected operations performed by experienced surgeons. As published in the *Annals of Surgical Oncology* by Henneman et al. [6], esophagectomies have better outcomes in centers with more than 20 cases per year. Another study showed that a surgeon reaches a plateau in thoracoscopic esophagectomy after 30 cases and better outcomes in terms of morbidity are seen after the 60th case [25].

Given the inconsistent conclusions in the previous studies, a multicenter study is required to achieve more precise results.

## Conclusions

In our study, we established that the surgeon’s experience might be considered a major risk factor in terms of developing CHT. This finding needs to be confirmed by a larger multicentric series taking into consideration the human factor.
